# Development and Industrial-Scale Fabrication of Next-Generation Low-Energy Membranes for Desalination

**DOI:** 10.3390/membranes12050540

**Published:** 2022-05-22

**Authors:** Li May Goh, Zhiwei Thong, Weikun Paul Li, Shu Ting Ooi, Farhanah Esa, Kok Seng Ng, Adil Dhalla, Chakravarthy Gudipati

**Affiliations:** Separation Technologies Applied Research and Translation Center (START), Nanyang Technological University—NTUitive Pte Ltd., Nanyang Technological University, Singapore 638075, Singapore; gohlimay@ntu.edu.sg (L.M.G.); thonhzhiwei@ntu.edu.sg (Z.T.); liweikunpaul@ntu.edu.sg (W.P.L.); shu-ting.ooi@outlook.com (S.T.O.); nur.farhanah@ntu.edu.sg (F.E.); ksng.mike@gmail.com (K.S.N.); adil.dhalla@ntu.edu.sg (A.D.)

**Keywords:** flat-sheet, spiral-wound modules, reverse osmosis, seawater desalination, reinforced membranes

## Abstract

Spiral-wound modules have been the most common configuration of packing flat-sheet membranes since the early development of polyamide (PA) membranes for water treatment applications. Conventional spiral-wound modules (SWMs) for desalination applications typically consist of several leaf sets, with each leaf set comprising feed spacers, membranes, and a permeate carrier (PC) wrapped around a permeate-collecting tube. The membrane area that can be packed into a given module diameter is limited by the overall leaf set thickness, restricting module productivity for a given membrane permeability. We describe here a novel industrial-scale method for successfully coating the polysulfone (PSf) ultrafiltration (UF) support layer directly onto a permeate carrier, instead of conventional non-woven fabric, as a precursor to the polyamide TFC coating, resulting in twofold benefits: (a) drastically simplifying the membrane fabrication process by eliminating the use of non-woven fabric and (b) increasing the throughput of each membrane module by facilitating the packing of a larger membrane area in a standard module housing. By combining the permeate carrier and membrane into a single sheet, the need for the non-woven support layer was eliminated, leading to a significantly reduced leaf set thickness, enabling a much larger membrane area to be packed in a given volume, leading to lower energy consumption per cubic meter of produced water. Molecular-weight cutoff (MWCO) values in the range of 36–96 kDa were found to be dependent on PC thickness and material. Nevertheless, the reinforced membranes were successfully fabricated with a ~9% reduction in membrane leaf thickness compared to a conventional membrane. Preliminary trials of coating a thin-film composite PA layer resulted in defect-free reverse osmosis (RO) membranes with a salt rejection of 94% and a flux of 40 L m^−2^ h^−1^ when tested against a 2000 mg/L NaCl feed solution at an operating pressure of 15 bar. Results from the testing of the 1812 and 2514 elements validated the novel concept and paved the way for further improvements towards full-scale RO membranes with the potential to be the next low-energy workhorse of the water industry.

## 1. Introduction

Reverse osmosis (RO) technology has remained the most dominant solution to water scarcity issues for the last few decades [1]. The ease of manufacturing and maturity of the thin-film composite coating (TFC) process have enabled the widespread commercialization [2] and adoption of RO technology with a comprehensive understanding of energy impact and desalination efficiency [3]. However, the RO process involves energy-intensive operation due to the high-pressure requirement to exceed the osmotic pressure [4]. Leon et al. analyzed the operational costs of seawater RO plants and reported that the energy consumed by high-pressure pumps forms about 62% of the total operational costs [5]. It has also been established that improving RO system efficiency by increasing permeability could reduce specific energy consumption, thereby leading to lower operating costs. Several research advancements have been made to reduce specific energy consumption (SEC) in the RO process for brackish water, as well as seawater applications. Efforts to improve process efficiency and lower SEC have been expended in different areas, including membrane structure, properties, operational process variables, and spiral-wound module components such as feed spacers. Some examples of more recent development towards lowering SEC in RO systems include (a) aquaporin-based biomimetic membranes, reported by Li et al. [6], which exhibited an 80% improvement in water flux compared to commercial RO membranes; (b) development of ultrahigh permeable membranes to improve module throughput in a given housing volume, as an approach to improve efficiency and reduce SEC [7]; (c) a seawater RO system (SWRO) in a hybridized operation with pressure-retarded osmosis and nanofiltration processes, in which net energy consumption was reduced to 1.4 kWh/m^3^ with a high recovery ratio of 88%, as reported by Touati et al. [8]; and (c) theoretical and experimental evaluation of commercial feed spacers with different geometries and permeability coefficients, to optimize SEC for brackish water [9] and seawater applications [10], as reported by Ruiz-Garcia et al.

Even though several researchers have reported significant developments in improving water permeability, at a given applied pressure, as an approach to achieving lower SEC at the lab-scale coupon level, few developments have been reported on the scaling-up of these membranes to spiral-wound modules for system-level testing or field validation. Spiral-wound modules are the only configuration currently in use in all RO-based water treatment plants. The objective to reduce the gap between the thermodynamic minimum for desalination (~1.1 kWh/m^3^) and the actual SEC in an RO process may never be realized, as it is constrained by engineering design limitations such as the efficiency of energy recovery devices or high-pressure pumps, also aggravated by process uncertainties such as fouling, scaling, and concentration polarization effects. Many such technical innovations involving high-permeable membranes, high-efficiency pumps, and energy recovery devices have been studied, as summarized by Voutchkov et al. [11]. Alternatively, process efficiency can be improved by significantly improving the permeate flux and product water recovery ratios in spiral-wound modules (SWMs). Cohen-Tanugi et al. reported that a threefold increase in permeate flux could effectuate a ~15% reduction in SEC [12]. Okamoto et al. reiterated a similar theory in their review, based on empirical evidence, that SEC can be lowered either by reducing hydraulic overpressure or by increasing permeate flux at a fixed applied pressure [13]. Very little has been published on work involving permeate carrier-based modifications to spiral-wound modules for improving module-level productivity as an approach to lowering SEC per unit volume of permeate water produced. She et al. reported the fabrication of non-woven fabric-reinforced membranes for pressure-retarded osmosis applications with water permeabilities up to 20 L m^−^^2^ h^−^^1^ bar^−^^1^ [14]. In another paper, Sun et al. reported the fabrication of fabric-reinforced aliphatic polyketone TFC membranes for pressure-retarded osmosis applications with a water flux of 24 L m^−^^2^ h^−^^1^ [15]. However, to the best of our knowledge, there are no reports of TFC PA membranes reinforced with permeate carrier materials for the optimization of permeate recovery to lower SEC in RO applications.

We report a novel method to optimize the membrane-spacer envelope in a spiral-wound module (SWM) to increase product water throughput at the module scale, to lower specific energy consumption (SEC) per unit volume of product water, in a desalination process. Towards this objective, we developed a novel process for the fabrication of a polysulfone (PSf) UF support layer directly on a permeate carrier mesh, instead of conventional non-woven fabric, followed by interfacial polymerization leading to a TFC PA layer, specifically for RO applications. Spiral-wound modules have been the most common configuration of packing flat-sheet membranes since the early development of polyamide (PA) membranes for water treatment applications. Additionally, the SWM configuration has long replaced the plate-and-frame configuration to become the only module configuration that is currently in use for flat-sheet RO membranes. Since the objective of this study is to demonstrate the feasibility of scaling-up novel reinforced membranes to the module level, we selected the standard spiral-wound module configuration for scale-up testing. A smaller leaf set thickness is critical for the fabrication of low-energy spiral-wound modules (SWMs) for many pressure-driven and thermal-driven membrane applications, such as reverse osmosis [16], membrane distillation [17], and pressure-retarded osmosis [18].

Even though current desalination technologies have matured immensely towards significantly lowering energy consumption from >7 kWh/m^3^ to ~3 kWh/m^3^, large-scale cost-effective desalination plants are urgently needed in several water-scarce regions. To expand commercial accessibility to SWRO desalination plants, it is necessary that desalination costs are reduced even further to make them affordable for developing nations. One approach to make the technology more accessible and deployable is to lower energy consumption costs, which account for more than 60% of operational expenses (OPEX) in desalination plants. Therefore, we propose a novel method to improve the efficiency of SWM RO modules by packing more membrane area. This results in higher product water per module at the same applied pressure, which translates to lower specific energy consumption per unit volume of produced water. The membrane fabrication method described in the current study involved reinforcing the membrane with a mesh material, such as a permeate carrier, to enable higher water permeability through a thinner support layer matrix, leading to lower resistance to permeate flow, as well as lower potential for concentration polarization [19]. This method offers another key advantage of replacing the typical non-woven substrate with a permeate carrier while retaining the permeate flow channels on the backside of the membrane in an SWM. This enables the membrane sheet to act as a separation barrier, as well as a permeate carrier, thereby significantly reducing leaf set thickness and facilitating more membrane area to be packed in a standard module. The packing of the larger membrane area in the same volume leads to higher product water output and, consequently, lower energy consumption per unit volume of produced water.

## 2. Materials and Methods

### 2.1. Materials

All chemicals used during membrane fabrication and scale-up were of industrial and reagent grade and used without further purification. Polysulfone (PSf) (BASF ULTRASON S 6010 NAT); lithium bromide (LiBr) (Sigma-Aldrich Pte. Ltd., Singapore); N-methyl-2-pyrrolidone (NMP) (MegaChem Ltd., Singapore); m-phenylenediamine (Sigma-Aldrich Pte. Ltd., Singapore); trimesoyl chloride (Sigma-Aldrich Pte. Ltd., Singapore); hexane industrial grade (MegaChem Ltd., Singapore); sodium chloride (NaCl) (Pure Dried Vacuum Salt, INEOS Enterprises, London, UK); permeate carrier of various mesh sizes (Shanghai Bright Imp. & Exp. Co. Ltd., Shanghai, China). Deionized water was acquired from a PURELAB Option-Q DV 25 unit from ELGA with a resistivity of 18.2 MΩ·cm.

### 2.2. Fabrication of Flat-Sheet Membranes

Flat-sheet membranes were initially fabricated with a formulation of the polymer dope solution for lab-scale casting developed by Tang et al [20] and scale-up casting conditions developed at our START center [21]. The casting dope formulation for industrial-scale casting was adjusted and optimized by START. The flat-sheet membrane substrate was fabricated via a non-solvent-induced phase-inversion method.

The industrial-scale casting was carried out using a slot die with a lip gap of 250 μm. The polymer dope was prepared in a jacketed polymer mixer and fed from the back of the slot die. Table 1 summarizes the casting parameters such as line speed and dope flow rate and includes the temperatures of the dope solution and coagulation bath. Briefly, the PSf dope was mixed separately in each batch for 24 h at room temperature: (1) was degassed for another 24 h in the jacketed reactor to guarantee complete dissolution of the polymer and removal of entrapped air bubbles in the mix; (2) the line speed of the substrate and the temperature of the coagulation bath were optimized during the casting process; (3) the flow rate of the wetting agent was optimized during casting; (4) after casting, the new membranes were rinsed in dechlorinated water to remove residual solvents; (5) the membranes were refrigerated to prevent the pore structure from collapsing; (6) a high-rejection polyamide coating was casted on the industrial-scale thin-film composite line; (7) the spiral-wound module was fabricated with a high-rejection reinforced membrane.

**Figure 1:**
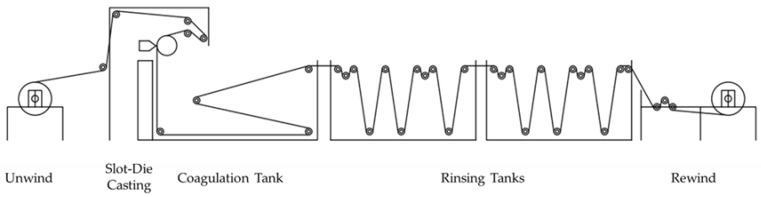
Schematic diagram of the pilot-scale phase-inversion casting line used in this study.

**Figure 2:**
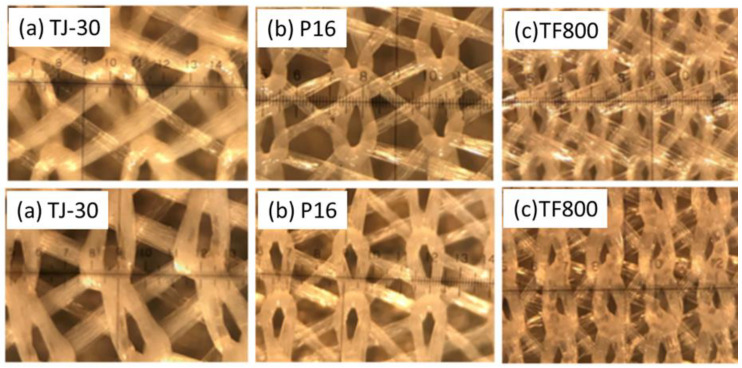
Optical microscope images of the reinforced materials used in this study: (**a**) TJ30 (pores ~ 400 μm), (**b**) P16 (pores ~ 300 μm), and (**c**) TF800 (pores ~ 100 μm).

As part of the initial scale-up trials, from the lab-scale fabrication line to the pilot scale of 1 m width (Figure 1), polymer, additive sources, backing material, solvents, and element rolling materials were sourced based on the cost of material and ease of availability for large-scale production. Several commercially available permeate carriers (PCs) were sourced for this study, in order to evaluate the effect of PC thickness and type of material on PSf membrane properties. The optical images of the different PCs used in this study are shown in Figure 2.

Different casting conditions were employed to optimize the membrane fabrication process, which can be scaled-up from small 1.5 kg batch sizes to 50 kg batch sizes. The coagulation bath temperatures, dope flow rate, and tensions were varied for the PSf material employed. The optimized conditions are shown in Table 1.

### 2.3. Characterization of Membrane Coupons

Optical images of the membranes were obtained using a Leica DVM6 optical microscope. Membrane morphologies were characterized with a field emission scanning electron microscope (FESEM) (JEOL JSM-7200F) operated at 5.0 kV of accelerating voltage.

The flux (L m^−2^ bar^−1^ h^−1^) of the high-rejection reinforced membrane coupons after interfacial polymerization were determined using cross-flow cells at room temperature using 2000 ppm NaCl solutions. To summarize, the flux of the membranes was evaluated using a transmembrane pressure, Δ*P*, of 15 bar and was calculated using Equation (1):

(1) $$Flux=\frac{Q}{A\Delta P}$$

where *Q* (L/h) is the water flux at the permeate side, Δ*P* (bar) is the applied transmembrane pressure, and *A* (m^2^) is the effective filtration area of the membrane. The membranes were allowed to stabilize for 1 h before any measurements were taken.

The concentrations of both the feed and permeate were evaluated using a conductivity meter (Lovibond SD 325 Con), and the effective rejection *R* (%) for each of the salts was calculated using Equation (2).

(2) $$R=\left(1-\frac{C_{p}}{C_{f}}\right)\times \;100\%$$

where *C_f_* and *C_p_* are the solute concentrations in the feed and permeate, respectively.

The molecular-weight cutoff (MWCO) of the porous reinforced PSf support layer was determined using solute rejection experiments. The MWCO is defined as the molecular weight of the solute at which the rejection is 90%. A feed solution consisting of dextrans with different molecular weights of 10 kDa, 40 kDa, 70 kDa, 500 kD, and raffinose pentahydrate was used in this study. During testing, a transmembrane pressure of 10 psi was used to induce the permeation flow across the fabricated membranes, as well as a series of control membranes that have known MWCOs with the feed solution. Prestabilization was taken before any measurement to minimize any dilution effects. A total organic carbon analyzer (Shimadzu TOC-LCPH-1202) was used to determine the solute concentrations in both the feed and permeate. The effective rejection coefficient *R* (%) for each organic solute was obtained using Equation (2). Subsequently, the solute rejection *R* of the controlled membrane was plotted against the known MWCO to yield a linear calibration curve. The MWCOs of the fabricated membranes were then obtained from the curve.

### 2.4. Spiral-Wound Module Testing

**Figure 3:**
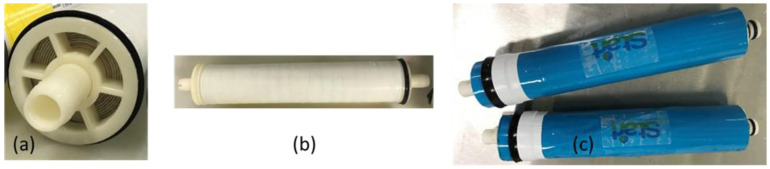
Examples of the 2514 (**a**,**b**) and 1812 (**c**) SWM elements that were assembled using the reinforced membranes coated with PA TFC layer.

The flat-sheet membranes prepared on the phase-inversion line were rolled into 1812 (1.8-inch diameter and 12-inch length), and 2514 (2.5-inch diameter and 14-inch length) spiral-wound modules (SWMs), as shown in Figure 3.

**Figure 4:**
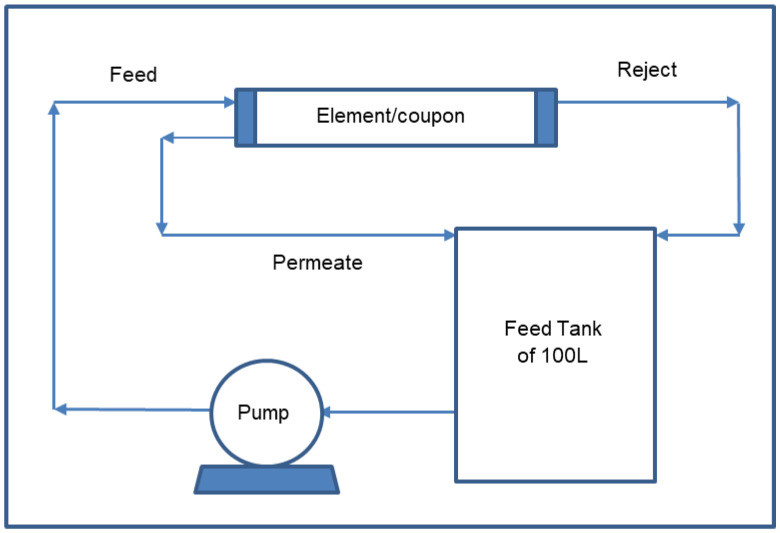
Schematic diagram of the setup used in this study to evaluate the 1812 and 2514 elements.

For the 1812 SWM, the elements were tested using a custom-built skid, the schematic of which is shown in Figure 4. The flux (L m^−2^ bar^−1^ h^−1^) of the membrane elements was determined using cross-flow element housing at room temperature using 500 ppm or 2000 ppm NaCl solutions at a pressure of 3.4 bar or 15 bar, respectively. The temperature of the feed was maintained using a chiller. Depending on the permeate flow rate of the elements, the feed flow rate was adjusted accordingly until the desired recovery was obtained. The recovery is defined as the ratio of the permeate flow rate over the feed flow rate as a percentage, as shown in Equation (3). The recovery of the 1812 element was kept at 15%, while that of the 2514 element was kept at 5%. These recoveries are in line with those of commercially available elements.

(3) $$\%\;Recovery=\left(\frac{Flow\;rate_{permeate}}{Flow\;rate_{feed}}\right)\times 100\%$$

## 3. Results and Discussions

**Figure 5:**
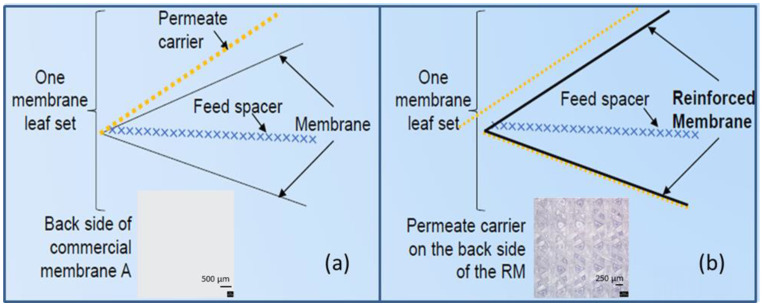
(**a**) Conventional spiral-wound module, which consists of 3 sheets, the feed spacer, the permeate carrier, and the membrane; (**b**) spiral-wound module with reinforced membrane consists of 2 sheets, i.e., the feed spacer and the reinforced membrane (combining the permeate carrier and membrane into a single sheet). The images of the backside of the membrane A and RM at magnifications of 200× the objective.

The conventional membrane-spacer envelope configuration in typical spiral-wound modules is shown in Figure 5a.

The reinforced membrane was cast onto the permeate carrier using a standard industrial-scale, 1 m wide, phase-inversion coater according to the process shown in Figure 1. In the current process, the phase-inversion method was slightly modified to pre-wet the permeate carrier, onto which the pre-mixed dope, with polysulfone (PSf) as the base polymer, was deposited using a slot die [20]. The typical dope formulation and the casting conditions are given in Table 1. The polymer cast substrate was passed through a coagulation bath maintained at room temperature at a line speed of 2.3 m/min. Polymer–solvent demixing and polymer phase-inversion resulted in UF support layer membrane formation on the substrate. During the preliminary trials in the current work, the reinforced UF layer was coated with a polyamide thin-film composite coating (TFC) using the formulation developed in our laboratory, to evaluate the integrity of the UF layer, as well as to validate the applications of these materials in different pressure-driven separations. Initial attempts at dope deposition and phase inversion directly on the permeate carrier with d (mesh size) > 300 μm produced membranes with pinhole defects and a weak polymer layer, which failed under high-pressure RO testing after interfacial polymerization (IP). Direct casting was successful on the permeate carrier with a smaller mesh size of d < 300 μm, but this method was not repeatable during scale-up to an industrial scale of 1 m width and more than 50 m length [22]. Additionally, permeate carriers with a smaller mesh size were either significantly more expensive or not readily commercially available. Another challenge encountered was to inhibit the polymer dope from seeping through the permeate carrier mesh and upon phase inversion, blocking the product water flow towards the permeate tube. In order to overcome this limitation, it was essential to pre-wet the mesh with a suitable wetting agent prior to casting the polymer and coagulation. The pre-wetting process involved the deposition of wetting agents such as the DI water, surfactants, and solvents on the permeate carrier as a primer before the PSf polymer coating, either on a hand frame or on the industrial fabrication line. This ensured the partial pre-coagulation of the PSf polymer instantly upon touching the wetted mesh, uniformly anchoring the polymer to the mesh surface, while the rest of the polymer phase separated inside the coagulation bath, resulting in defect-free membranes devoid of pinholes while also preserving the permeate channels for product water flow during RO operations. In the lab-scale hand-frame casting, in addition to ensuring the thin coagulated layer formed instantly after casting onto the wetted mesh, the phase-inversion process was carried out with the polymer surface facing down as the weight of the casted polymer pulled the polymer away from the mesh and prevented the dope from seeping through the holes. The coagulating membrane facing upwards was not as robust, as the weight of the polymer pulled the polymer through the holes and had a high probability of creating a pinhole defect or blocked permeate channels.

**Figure 6:**
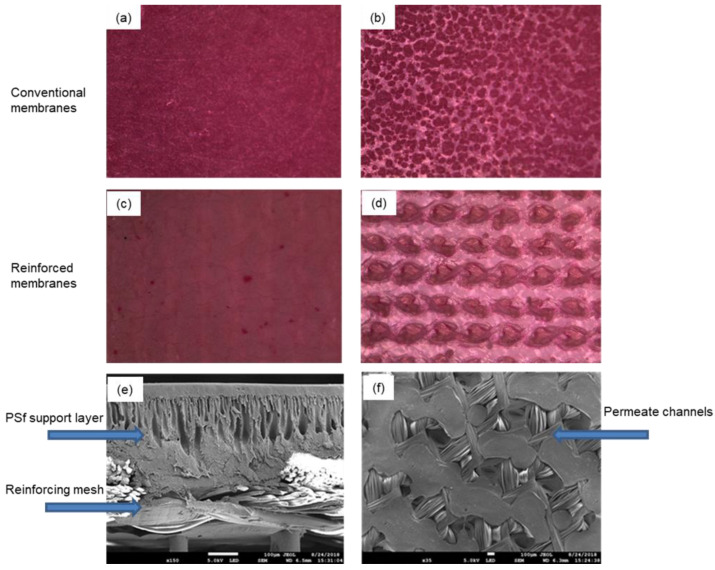
Optical microscope images of the front (selective layer) and back (support layer) surfaces of the reinforced membrane coated with polyamide thin-film composite coating on a conventional non-woven polyester backing (**a**,**b**) and on a TJ-30 permeate carrier (**c**,**d**). (**e**,**f**) FESEM images of the cross-section and the back side of the reinforced membrane, respectively.

Figure 2 shows the optical microscope images of all the permeate carriers that were used in this study, along with the mesh sizes. Permeate carriers with a variety of mesh hole sizes ranging from 100 microns to 400 microns were employed to demonstrate the versatility of the fabrication method. This lab-scale coating method with pre-wetting was scaled-up and translated successfully onto the industrial-scale coating line for fabricating the reinforced membrane. On the industrial-scale coating line of 1 m width and length > 50 m, the PSf polymer was cast using a slot die positioned perpendicular to the substrate (Figure 1). Due to the higher porosity of the mesh as compared to a non-woven polyester backing, the casting process was challenging due to significant shrinkages and creasing along the cast membranes, resulting from in-line stress due to continuous operation of the line. The process was modified to adjust the line speed and, consequently, the line tensions so as to mitigate the creasing and the in-line bending of the web. This ensured that the process was stabilized and optimized over several rounds to achieve a good substrate with no pinhole defects across the 1 m width. Figure 6 shows the front and back view of the conventional flat-sheet RO membrane (Figure 6 a,b) and the newly fabricated low-energy membrane (Figure 6 c,d) with a polyamide selective layer. It can be easily seen that, unlike the regular flat-sheet membrane, the newly fabricated membrane had clearly defined water channels on the back, as also confirmed by the FESEM images of the membrane cross-section (Figure 6e), as well as the backside of the membrane (Figure 6f). Therefore, the reinforced membranes have the potential to replace the typical non-woven substrate with the permeate carrier and enable single-membrane sheet element rolling with feed spacer, which then could significantly reduce the cost and labor for element rolling. The membrane when rolled into a standard module would require lower energy for the same output as more membrane could be rolled within the standard housing.

**Figure 7:**
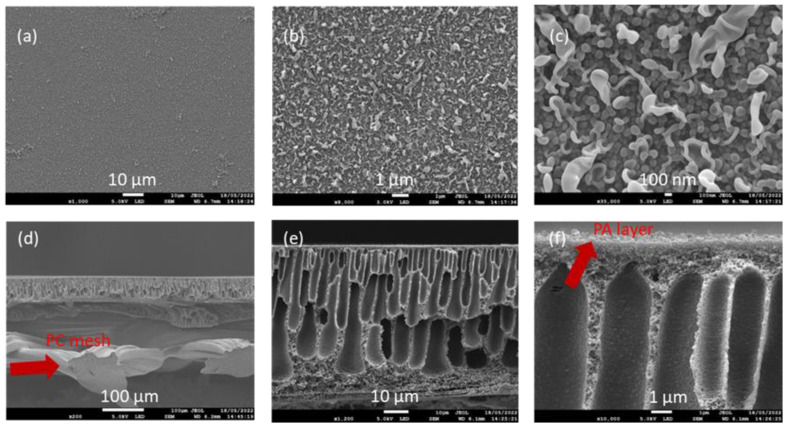
FESEM images of the reinforced membranes after TFC PA layer coating. (**a**–**c**) Top surface of the membrane depicting the PA layer coating, (**d**) membrane cross-section images showing the reinforcing permeate carrier material, (**e**) UF support layer with finger-like macro voids, and (**f**) zoomed-in image of the UF support layer with the PA layer.

The reinforced PSf support layer was coated with the polyamide TFC coating through IP on an industrial coater to make the dense selective polyamide (PA) layer. Figure 7 depicts the FESEM images of the reinforced membrane after the TFC PA layer coating. A continuous nanoporous “ridge-and-valley” morphology, which is typical of the TFC PA layer [23], is clearly seen on the surface of the membrane, as shown in Figure 7a–c, which confirms the formation of the TFC selective layer. The cross-sectional images (Figure 7d–f) showcase the presence of the permeate carrier material impregnated in the polymeric matrix (Figure 7d), while the UF support layer filled with finger-like macro void morphologies is shown to be preserved. The membrane was then wound into spiral-wound modules (Figure 3), with the feed spacer facing the active selective layer side and the permeate carrier facing the backside of the membrane. The method here originated from the key objectives of fabricating a pressure-driven reinforced membrane for pressure-driven application and with a thin leaf set with permeate carrier and membrane combined in a single sheet. This thin leaf set was critical for the fabrication of low-energy spiral-wound modules (SWMs) for all pressure-driven membrane applications and enabled two-layered membrane sheet element rolling as compared to the typical three-layer rolling (Figure 5).

TJ-30, with the largest average mesh hole size of 400 microns, was selected to demonstrate the effect of pre-wetting, as well as the pre-wetting solution, on membrane performance (Table 2). The pre-wetting of the permeate carrier was conducted prior to the casting of polymer solution to prepare the substrate membrane. Subsequently, a thin-film composite (TFC) polyamide selective layer formed on the resultant substrate membrane via interfacial polymerization. It can be seen from Table 1 (Entries 5 and 6) that without any pre-wetting with water or other agents, the substrate membrane was highly defective, and it was highly difficult to form a defect-free polyamide selective layer. This was presumably because, without pre-wetting, the PSf polymer solution intruded into the mesh holes upon deposition, leading to non-uniform surface coverage on the mesh, which resulted in a defective PA layer, as seen in Table 1, likely because the pre-wetting method helps to fill up the mesh holes of the permeate carrier prior to coating. The pre-wetting process temporarily sealed the mesh pores with pre-coagulation of the PSf polymer and eliminated the problem of solution intrusion. As a result, the flux and rejection of the TFC membrane could be further improved (Table 2).

Next, the effect of the type of pre-wetting solution on the membrane performance was explored. Generally, the permeate carriers are made of polyester, which tends to be hydrophobic. Thus, for the hydrophilic pre-wetting solution of water or aqueous agents to fill the mesh holes more easily and completely, the weight percentages of sodium laureth sulfate (SLS) or N-methyl-2-pyrrolidone (NMP) in the pre-wetting solution were increased. Interestingly, it was found that increasing the SLS content to 0.2 wt.% or the NMP content to 20 wt.% had a negligible impact on the performance of the membrane. When further increasing the SLS content to 2.5 wt.% or the NMP content to 80 wt.%, the membrane performance deteriorated drastically, leading to a significant loss of salt rejection. This was likely because the highly hydrophilic pre-wetting solution may have interfered with the phase-inversion process of the polymer dope and prevented the formation of a defect-free substrate.

In general, as the mesh size increases, the MWCO is also expected to increase as the PSf solution intrusion into the mesh hinders the formation of a homogenous UF support layer during the phase-inversion process. However, in our study, no clear trend was observed for the relationship between the MWCO data and the mesh size (Table 3). In comparison to the control membrane (non-woven), TF800, with the smallest mesh size, gave the smallest MWCO of 37 kDa, presumably due to lower solution intrusion and the formation of a thicker substrate layer. On the other hand, the MWCO increased to 96 kDa for the P16 material with a mesh size of 300 μm, which reduced to 54 kDa for the TJ030 material. Given the smaller scale of experiments, several factors, including mass transfer through the mesh during phase inversion, as well as other extraneous factors such as variations in dope viscosity through solvent evaporation or moisture, may have contributed to this observation.

Table 3 demonstrates the effect of the average mesh hole size on the membrane performance. The water fluxes for the UF support layer membranes prepared using conventional non-woven fabric, TJ-030, P16, and TF800 were measured as 130 ± 18 L m^−2^ h^−1^, 136 ± 36 L m^−2^ h^−1^, 115 ± 36 L m^−2^ h^−1^, and 186 ± 44 L m^−2^ h^−1^, respectively. It can be observed that as the average mesh hole size increases from 100 microns to 400 microns, the average flux of the resultant TFC membrane decreases from 48.65 L m^−2^ h^−1^ to 21.95 L m^−2^ h^−1^, while the NaCl rejection remains relatively constant at 95%, for all the pre-wetted substrates. This was likely because as the average mesh hole size increased, the amount of solution intrusion into the mesh hole increased as well. Hence, a greater amount of polymer needed to be deposited to achieve a defect-free substrate, and as a result, the permeate transport resistance of the resultant membrane increases. In addition, it was also observed that there was an optimum mesh hole size of about 300 microns (P16), which enabled the greatest reduction in thickness compared with the non-woven membrane backing while having comparable membrane performance. Thus, Permeate P16, with a ~9% reduction in thickness, shall be used for further scale-up on the industrial fabrication line.

Table 4 demonstrates the successful translation of the fabrication method onto the industrial line, which was 1 m in width with ~7.4% reduction in thickness compared to the conventional membrane. The performance of the line run membrane with a flux of 51.1 L m^−2^ h^−1^ and 2000 ppm NaCl rejection of 95.9% is comparable to that of the hand-frame membrane, which has a flux of 42.1 L m^−2^ h^−1^ and 2000 ppm NaCl rejection of 94.8% Subsequently, the 1812 (1.8-inch diameter and 12-inch length) and 2514 (2.5-inch diameter and 14-inch length) elements were fabricated from the above-mentioned membrane. The 1812 element has a flux of 0.032 L per min (LPM) and a 500 ppm NaCl rejection of 79.45% under the operating pressure of 3.4 bar, while the 2514 element has a flux of 0.325 LPM and a 2000 ppm NaCl rejection of 87.25% under the operating pressure of 15 bar. Successful industrial fabrication and demonstration of next-generation low-energy membranes have been achieved.

## 4. Conclusions

- We successfully developed a novel process to fabricate reinforced flat-sheet membranes on permeate carrier materials, instead of conventional non-woven fabric materials.
- The reinforced membrane fabrication process can be translated to an industrial-scale fabrication line for large-scale manufacturing.
- Through preliminary trials, we demonstrated that the reinforced PSf support layer is amenable to interfacial polymerization, leading to a defect-free thin-film composite polyamide coating with high flux and salt rejection.
- Preliminary experiments successfully validated the concept of reinforced membrane fabrication on the industrial-scale membrane production line and will pave the way for large-scale production once the proof-of-value is established through large-scale pilot testing.
- A very high flux of 65 LMH and >90% salt rejection was observed for the RO membranes prepared on the TF800 permeate carrier with a mesh size of 100 μm. While further study using the TF800 was limited by the high price and availability of the materials, cheaper source identification and process optimization trials will ensue in future studies.
- The TFC coating on the reinforced membranes resulted in a flux comparable to commercial RO membranes (1–1.5 LMH/bar) and rejection of up to 95 % on coupon-level testing and up to 87% on 2514 element testing.
- An overall membrane leaf set thickness reduction of 8–10% was successfully achieved, and the resulting free volume enables the packing of a much larger membrane area in a given volume in commercial modules (1812, 2514, 4004, or 8040).

The objective of this study was to demonstrate the proof-of-concept demonstration of fabricating PSf UF support layer membranes impregnated with permeate carrier mesh material. The strategy was to reduce membrane leaf set thickness in order to improve membrane efficiency at the module level by packing more membrane surface area in each element housing, resulting in overall increased product water output. Increased output per module translates to lower energy consumption per cubic meter of the RO permeate. Further research is ongoing to optimize the TFC coating procedure on the fabrication line, along with the optimization of module configuration to achieve better or comparable performance of commercial RO membranes (0.7–1.0 LPM flux and >98% rejection) and will be the subject of a subsequent full publication. The high flux and relatively lower rejection, compared to commercial RO membranes, leaves a lot of room for improvement of the membrane characteristics. Nevertheless, the preliminary trials resulting in defect-free TFC RO membranes validate the concept of reinforced membranes, which can be manufactured on a large scale. The TFC chemistry, once optimized [24], will pave the way for the large-scale manufacturing and adaptation of next-generation RO membranes for brackish water and seawater desalination, with much higher system-level efficiency and product water recovery at the same pressure, leading to net-lower SEC. This is beneficial to mankind not just from a water scarcity point of view by lowering desalination costs, but also by leading to lower emissions towards a low-carbon future.

## Figures and Tables

**Table 1:** Casting conditions for industrial-scale production of reinforced flat-sheet membranes.

Phase-Inversion Line Conditions	
Dope solution	PSf/LiBr/NMP
(wt. %)	17/2/81
Line speed (m/min)	2.3
Dope flow rate (mL/min)	500
Pre-wetting solution	DI
Pre-wetting solution flow rate (mL/min)	300
Dope temperature (°C)	Room temperature (~20)
Coagulation bath temperatures (°C)	Room temperature (~20)

**Table 2:** Effect of pre-wetting on the RO flux and salt rejection of membrane, using different wetting agents. The RO testing was performed using 2000 mg/L NaCl solution at 15 bar pressure.

No.	Membrane Backing	Pre-Wetting Solution	Flux (L m^−2^ h^−1^)	Rejection (%)
1	TJ-030	2.5 wt.% SLS	Very high	Negligible
2	TJ-030	0.2 wt.% SLS	29.2	82.8
3	TJ-030	80 wt.% NMP, 20 wt.% water	89.1	25.9
4	TJ-030	20 wt.% NMP, 80 wt.% water	27.9	93.6
5	TJ-030	DI	22.0	95.0
6	TJ-030	None	Very high	Negligible
7	Non-woven fabric	None	25.8	98.2

**Table 3:** Effect of average mesh hole size on the membrane performance. The membrane performance was evaluated with a pressure of 15 bar and 2000 ppm NaCl solution. Negative sign indicates reduction in the film thickness. The MWCO data were measured on the reinforced PSf membrane layer.

Membrane Backing	Average Mesh Hole Size	Pre-Wetting Solution	Flux (L m^−2^ h^−1^)	Rejection (%)	MWCO (kDa)	Change in Membrane Thickness (%)
TJ-030	≈400 microns	DI	21.98 ± 4.10	95.0 ± 2.7	53.9	−1.4%
P16	≈300 microns	DI	40.97 ± 3.80	94.1 ± 9.5	96.0	−8.6%
TF800	≈100 microns	DI	65.28 ± 6.11	91.6 ± 10.5	36.9	−5.8%
Non-woven	NA	None	25.78 ± 2.78	98.2 ± 0.9	54.0	0%

**Table 4:** Industrial demonstration of the fabrication method and performance of the commercial-sized membrane elements. (3.4 bar, 500 ppm NaCl) (15 bar, 2000 ppm). Negative sign indicates reduction in thickness compared to the non-woven substrate-based membrane.

/			Coupon Testing		1812 Element		2514 Element	
Membrane Backing	Pre-Wetting Solution	% Change in Thickness	Flux (L m^−2^ h^−1^)	Rejection (%)	Flux (LPM)	Rejection (%)	Flux (LPM)	Rejection (%)
P16	DI	−7.37%	40.97 ± 3.76	94.1 ± 9.5	0.032 ± 0.012	79.5 ± 10.1	0.279 ± 0.054	87.7 ± 5.4
Non-woven	None	0%	25.78 ± 2.78	98.2 ± 0.9	NA	NA	NA	NA

## Data Availability

The data presented in this study are openly available DR-NTU (Data) at https://doi.org/10.21979/N9/UIEEDQ.
